# Assessing Ulcerative Pododermatitis of Breeding Rabbits

**DOI:** 10.3390/ani3020318

**Published:** 2013-04-17

**Authors:** Joan M. Rosell, L. Fernando de la Fuente

**Affiliations:** 1 Cunivet Service, P.O. Box 518, 43080 Tarragona, Spain; 2 Departamento de Producción Animal, Facultad de Veterinaria, Avda. Profesor Pedro Cármenes s/n, Universidad de León, 24071 León, Spain; E-Mail: f.fuente@unileon.es

**Keywords:** animal welfare, disease prevention, footrest, rabbit, sore hocks, ulcerative pododermatitis

## Abstract

**Simple Summary:**

Sore hocks are a health and welfare problem in rabbits housed in cages with mesh floors. Footrests are used to prevent them. They occupy part of the mesh floor of the cage but allow droppings to fall and also protect the rabbit’s feet. In this study we evaluated the use of footrests on 664 commercial farms visited in Spain and Portugal, and the rates of sick animals during 2001–2012; the attention given by producers to animal care was evident as 28% of farms with footrests in 2001 increased to 75% in 2012.

**Abstract:**

Rabbits in conventional farms are housed in wire net cages with mesh floors to separate them from droppings. In time, lacerations appear on the legs of adult rabbits causing ulcerative pododermatitis or sore hocks, a severe health and welfare problem. Pain causes behavioral changes; productivity is reduced and the most seriously affected animals die or are culled. In this study we evaluated the attention producers have given to this problem and its prevention by installing footrests in cages. We made 2,331 visits to 664 commercial farms in Spain and Portugal between 2001 and 2012, and evaluated morbidity by examining 105,009 females and 10,722 males. The study highlights that the rate of farms with footrests increased from 27.8% in 2001 to 75.2% in 2012. Prevalence of sore hocks in does in 2001 was 11.4%, decreasing to 6.3% in 2012; prevention of ulcerative pododermatitis was associated (*P* < 0.001) with the presence of footrests. Overall, prevalence was 4.87 ± 0.26 on farms with footrests and 13.71 ± 0.32 without (*P* < 0.01).

## 1. Introduction

Rabbits in conventional farms are housed in wire net cages with mesh floors; these separate them from droppings and maintain a certain level of hygiene [1]. An adult female or male rabbit lives under these conditions, until it dies or is culled; the median culling age per doe is 14.9 months old and 17 months old per male [2]. The existence of lesions in the plantar region of the limbs, named sore hocks or ulcerative pododermatitis [3], was evident since rabbits were reared on this type of floor [4]. Sore hocks are typically influenced by the environment [5], as is lameness in milking cows [6]. This condition causes chronic pain and suffering [7]. Breeding rabbits with footpad injuries have reduced ability to walk or stand [8], and might be anorexic [9]; sick rabbits have a poor body condition [10]. Productivity is reduced: loss of male libido, low fertility of does and viability of their kits are observed. Life expectancy of rabbits with sore hocks decreases [2]; in fact, from the logic of the *five freedoms*, this disease causes poor welfare [11].

As rabbit production became more intensive, the technical and financial importance of this type of injury and leg disorder grew [5]. Thus, veterinarians practicing on rabbits included sore hocks in morbidity targets. During 1986–1996, after examining 103,968 does and 15,987 bucks in 3,276 samplings on 762 farms, the mean prevalences of sore hocks were 9.1% and 7.5% in females and males, respectively [12]; these were the baselines of prevalence we used when examining adult rabbits, though other authors have found higher prevalences: 12% [13].

Between 1990 and 2000, several studies were carried out on the effects of different types of floors on foot lesions [14,15], and the prevention of sore hocks by using plastic mats [16]; their efficacy was scientifically proven, and also from empirical perspective, as technicians informed producers or evaluated tests carried out in farms and, as a result, morbidity decreased [17]. However, more science-based information on rabbit housing is necessary [18]. 

Our aims were to (1) determine the evolution of the use of footrests as a mean for improving flooring in breeder cages, and (2) assess their effect on the prevalence of sore hocks, during 2001–2012.

## 2. Material and Methods

### 2.1. Population Description and Sampling

From January 2001 to December 2012 we gathered information on the use of footrests during 2,331 visits to 664 farms: 610 farms in Spain and 54 in Portugal. There were does on 635 farms and males on 182 farms, including 29 Artificial Insemination (AI) centers. All of these visits were carried out by a single, trained veterinarian (Rosell). The objective of every visit was to assist the producers in case of emergency or to assess technical and economical efficiency of rabbit farms, in the absence of a specific disease outbreak in the herds. Protocol used during the visits included, firstly listening the history and the producer’s opinions; right after, the study of the production records. Besides, we observed the environment, e.g., on-farm climate (mainly: temperature, humidity and air speed), the cages (*i.e.*, dryness of floor, rusty; presence of footrests and their conditions: cleanliness, eroded, broken); and other husbandry traits (e.g., watery and feeding systems, breeding management). The visit also comprised the fitness assessment of breeders and kits, by examining the body condition and morbidity of coryza, mastitis, sore hocks and manges, and to perform necropsies of sudden dead or currently ill-moribund rabbits, as main diagnostic tools [19]. During each visit, some water feed or pathological samples were taken. Finally, we concluded the visit by writing a summary. Given that data were gathered by a veterinary practitioner during visits to rabbitries, they do not follow an optimally balanced design: of the total 2,331 visits to 635 doe farms during 2001–2012, we examined females only on 413 doe farms (65%). Two-hundred and one doe farms of the 413 we examined (48.6%) were sampled once, 69 twice, 43 three times, 77 farms 4 to 12 times, 16 farms 13 to 24 times, and 7 were sampled more than 24 times in the course of 12 years.

### 2.2. Sampling Protocol

In this study there was information obtained during a monitoring process on the farms [20], such as we recorded whether the does, males, or both, had footrests or not; we used a binary variable. When we needed information on body condition and morbidity caused by sore hocks, we examined lactating does or males, approximately a 10% from these breeders, including 10% of primiparous females of the batch; in a previous study [10], it was shown that this type of sampling guarantees representativeness of all the females on the farm. In view of the dispersion in the size of the sampled doe farms: median 400 does at risk, ranging from 40 to 3,000 females, the number of sampled animals per visit varied, ranging from 10 to 219 does, with a median of 60 does. Concerning males, the median size of the 162 sampled male farms, including 24 sampled AI centers, was 60 males at risk (minimum to maximum: 8–544 males), with a median of 15 males per sample, ranging from 8 to 100 males. There was also information related to the surveillance [20] on the farms, because we recommended and explained actively the use of footrest to rabbit producers.

### 2.3. Assessment of Morbidity

A binary variable indicating whether the animal had or did not have clinical signs for ulcerative pododermatitis was defined. A rabbit has sore hocks when a plantar or volar lesion is observed on at least one limb, as can be seen in the pictures of a link provided by Rosell [21], even the first stage; however, in our practice we did not use score grades for assessing ulcerative pododermatitis. In our protocol, we did not consider hyperkeratosis to be a lesion, neither did other authors [22], unlike Drescher and Schlender-Böbbis’ criterion [23]. Nevertheless, a callus might indicate the risk of a lesion occurring or, on the other hand, of healing [24]. We also differentiated sore hocks in the plantar region of the hind limbs or in the volar region of the front limbs, from digital dermatitis and other cutaneous manifestations, due to foot-pad pseudomonosis, ringworm or sarcoptic mange. Disease occurrence was recorded through prevalence [25].

### 2.4. Statistical Analysis

The dependent variable was the prevalence of sore hocks (PSH). We measured it based on the percentage of affected does, in comparison with the population at risk on the day of the visit. Statistical analysis was utilizing GLM procedure of SAS (SAS Inst., Inc., Cary, NC, USA). Statistical significance was indicated by a *P* < 0.05. The unit of analysis was the farm. The statistical model used was the following:

Y_ij_ = μ + YE_i_ + FR_j_ + RA_k(j)_ + e_ijk_

where Y_ij_ was the dependent variable PSH on each visit, μ was the mean, YE_i_ was the year effect (2001–2012), FR_j_ was the footrest effect, RA_k(j)_ was the rabbitry effect, and e_ijk_ was the residual effect. 

## 3. Results and Discussion

### 3.1. Description of the Data

The characteristics of farms housing breeder rabbits are shown in Table 1. The 635 doe farms visited during 2001–2012 had a global median of 680 females per farm, which corresponds to the most specialized segment of 2,100 farms housing >200 females, according to the National Rabbit Breeding Survey [26]. 

**Table 1:** Number and traits of the farms visited in Spain and Portugal during 2001–2012.

Year	2001	2002	2003	2004	2005	2006	2007	2008	2009	2010	2011	2012	Global
Visited farms	107	97	99	163	144	148	159	169	184	110	147	149	664
Total visits	125	113	106	249	188	178	213	243	301	137	234	244	2,331
Visited doe farms	98	93	94	152	143	143	151	164	171	102	140	141	635
Present does (total)	83,278	91,991	77,170	133,874	133,365	120,496	157,070	150,405	150,429	102,730	142,686	129,374	555,966
Median of does/farm	600	688	600	596	714	675	750	713	744	700	825	735	680
Minimum of does Maximum (per farm)	120 4,500	102 6,000	102 6,000	75 6,200	75 5,000	68 4,880	72 5,250	98 5,825	40 4,000	100 10,000	96 10,000	70 7,500	40 10,000
Visited male farms (AI centers)	63 (9)	40 (4)	31 (5)	56 (11)	47 (1)	40 (5)	37 (8)	23 (5)	41 (13)	40 (8)	39 (7)	40 (8)	182 (29)
Present males (total)	4,469	3,194	2,987	3,626	3,331	3,370	3,473	2,430	4,703	4,926	4,491	4,520	14,650
Median of males per farm (range)	50 (15-400)	60 (8-500)	70 (12-500)	50 (12-300)	50 (12-250)	52 (12-530)	60 (12-400)	70 (24-440)	72 (15-600)	68 (20-585)	61 (12-428)	70 (10-800)	45 (8-800)

The last column shows the total number of farms visited (664), how many housed females (635), only females (482), males (182), only males in 29 AI centers, or both females and males (153). 

Table 2 shows the results of the analysis of variance, corresponding to the model explaining prevalence of sore hocks (PSH), showing the significance of the different factors in the trait.

**Table 2:** The ANOVA for the Prevalence of Sore Hocks in rabbit females.

Source of variance	Df	F-Value	Pr > F	% variance
**Year**	11	2.02	*	0.45
**Footrest**	1	512.64	***	53.46
**Rabbitry (Footrest)**	530	2.81	***	8.94
**Model**	542	4.56	***	62.86

* *P* < 0.05; *** *P* < 0.001

The proposed model of ANOVA included most of the variability of PSH character: 62.86%. The highest influencing risk factor in the variability of PSH (53.46%) was footrest (Yes *vs.* Not footrest). The second most important factor was rabbitry (8.94%), which included the associated factors of housing and husbandry. These results show the great influence of the footrest on the prevalence of sore hocks.

### 3.2. The Use of Footrests during 2001–2012

**Figure 1:**
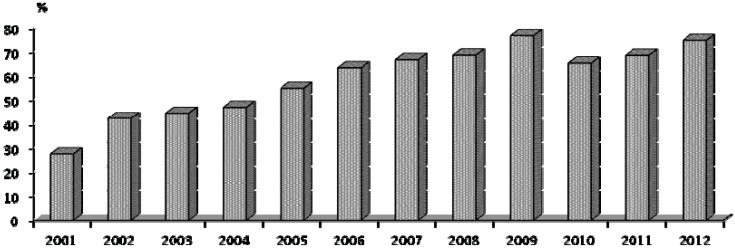
Evolution of the percentage of visited farms using footrests. There were 635 doe farms visited in Spain and Portugal during 2001–2012.

Figure 1 shows the evolution of the percentage of farms with footrests, which increased from 27.8% in 2001 to 75.2% in 2012. From the database, we took a subgroup of 37 farms visited both in 2001 and 2012, to determine their evolution; in 2001, 17/37 (46%) of these farms had footrests in comparison with 33/37 (89.2%) in 2012. These findings may be highlighted in our study.

The global database corresponding to the 635 doe farms and the fixed sample of 37, show that technical progress (cages with footrests) has clearly been made, despite the two drawbacks observed by producers: the price of the footrest (for example, 1.2 Euros in 2013, for a plastic mat measuring 37 × 24 cm, manufactured by Gómez and Crespo, Ourense, Spain [27]), and occasionally, the work involved in cleaning or replacing it; these are the arguments made by producers who do not use footrests [28]. 

### 3.3. Evolution of Morbidity

By evaluating morbidity we aimed to determine part of the possible cause/effect relationship. The decrease in morbidity of sore hocks is very evident. During 2001–2012 we examined 105,009 lactating does in 1,603 samplings on 413 rabbitries, housing females or females and males, as well as 10,722 males (533 samplings) on 162 buck farms: 138 with does and males, and 24 AI centers. Mean prevalences were 7.5% and 5.2%, in females and males, respectively; we can observe the evolution of these results, with 9.1% (females) and 7.5% (males) in 1986–1996. In 2001, prevalence in females was 11.4%, whereas in 2012 it was 6.3%. Table 3 shows the least square means of prevalence of sore hocks (PSH) and also their statistical significance. 

**Table 3:** Least squares means and standard errors for the footrest effect. There were 1,603 samplings and 105,009 lactating females examined on 413 doe farms, during 2001–2012.

Footrest (samplings)	Prevalence. Mean ± SE
Yes (1,121)	4.87 ^a^ ± 0.26
No (482)	13.71 ^b^ ± 0.32

^a^^,b^ Means in the same column with different superscripts differ (*P* < 0.01).

In our database, the mean prevalence of sore hocks in females with footrests was 4.9%, in comparison with 13.7% in those without. The decrease in morbidity due to the use of footrests may also be highlighted in the study; rabbits can easily cope with a relevant part of their environment, such as the enriched cage flooring [29]. Based on this, we may state that footrests should be included in disease prevention programs, in agreement with Cockram and Hughes [30]. Nevertheless, we believe that a more in-depth analysis of the prevalence of sore hocks on the basis of several predisposing risk factors, such as the age or the genetic type, as well as other enabling risk factors [25], besides the footrest, is necessary, and might be the subject of future studies.

### 3.4. Implications

Firstly, this study shows that rabbit producers have already installed footrest in the breeding cages, during the evaluated years. From a welfare perspective, footrests enable the rabbits to cope with their environment [29]. From an epidemiological point of view, the present study contributed also to assess a part of rabbit health: it showed that prevalence of sore hocks have decreased, similarly as the decline in incidence risk [28]. This study highlights that the partial enrichment of the rabbit environment with the footrest, is a practical strategy for improving rabbit health and welfare, as quoted also above [10]. This progress in medical knowledge has certain importance [31]. Secondly, producers view the improvement positively from the technical and financial perspective [32]. Thirdly, by including footrests in the cages, producers have already improved the rabbit care, [33], a key aspect for a quality assurance scheme in animal production [34]. Fourthly, this change might be related to the sustainability of rabbit production [35]. Finally, from the perspective of external assessors, in particular veterinarians, which one key function is to assess animal health and welfare, this enhancement is an incentive to continue working [36]. 

## 4. Conclusions

Our aims in this study were, on one hand, to assess the welfare conditions of rabbit farms, such as the use of footrests in cages over the course of years and, on the other hand, to assess rabbit health, such as the prevalence of ulcerative pododermatitis in breeding rabbits.

The observations made during 2,331 visits to 664 rabbitries between 2001 and 2012, show that rabbit producers have given particular attention to the problem of ulcerative pododermatitis and its prevention. We related the progress in health found after examining 105,009 does and 10,722 bucks, to this practice. However, in the future our aim will be to evaluate further risk factors of the disease.

Health and welfare are key aspects of sustainability in rabbit production, as is the case in other domestic species [37,38]; therefore, if in the future rabbits will be housed in cages with mesh flooring, they should also have footrests as an essential part of their design, of disease prevention programs, and a quality assurance scheme. With this in mind, we must show the scientific evidence found and enhance the perception of risk maintained by rabbit producers who have not yet installed footrests. 
